# Breakthrough Infections: Clinical Profile and Outcomes of COVID-19 Vaccinated and Unvaccinated People From a Tertiary Care Hospital

**DOI:** 10.7759/cureus.32089

**Published:** 2022-12-01

**Authors:** Praveen R Shahapur, Roopa Shahapur, Smitha Bagali, Rashmi Karigoudar, Dr Sanjay Wavare, Jyothi P, Venkataramana Kandi, Tarun Kumar Suvvari, Rahul J Mittal, Mamtha Jadhav

**Affiliations:** 1 Microbiology, Shri B.M. Patil Medical College Hospital and Research Centre, BLDE (DU), Vijayapura, IND; 2 Dentistry, Shri B.M. Patil Medical College Hospital and Research Centre, BLDE (DU), Vijayapura, IND; 3 Clinical Microbiology, Prathima Institute of Medical Sciences, Karimnagar, IND; 4 Medicine and Surgery, Rangaraya Medical College, Kakinada, IND; 5 Medicine, Gujarat Medical Education and Research Society Medical College, Gandhinagar, IND; 6 Medicine, Jawaharlal Nehru Medical College, Datta Meghe Institute of Medical Sciences, Wardha, IND

**Keywords:** clinical profile, outcomes, unvaccinated, vaccinated, vaccination, coronavirus disease-19, breakthrough infect

## Abstract

Introduction

Despite the availability of a vaccine and extensive vaccination, breakthrough infections are commonly noted, which is jeopardizing the vaccine-based protection against COVID-19. The present study aims to evaluate COVID-19 breakthrough infections and to compare the clinical profile and outcomes of the vaccinated and unvaccinated populations.

Methods

A retrospective observational study was conducted for two months (March-April 2021), and all cases reported during the study period were included in the study. Socio-demographic details, COVID-19 profiles, clinical outcomes, vaccination statuses, and types of vaccine were collected from the patients. Further, COVID-19-positive samples were screened for lineages using next-generation sequencing (NGS).

Results

Of the total 103 patients included in the study, 79 (76.7%) were symptomatic and 24 (23.3%) were asymptomatic. Only 32% were vaccinated and 68% were unvaccinated. 29.2% were hospitalized due to COVID-19 and all of them were unvaccinated. The mortality among hospitalized patients was extremely high (60%). The time to positivity after complete vaccination was noted to be 37.09±23.74 days. The unvaccinated study participants showed lower Cycle threshold (Ct) values (E Gene/N Gene: 17.38±4.53) as compared to the vaccinated people (E Gene/N Gene: 22±4.25). The Delta (B. 1.1. 629) (76.7%) was the predominant variant among the study population followed by AY.4 (20.4%) and Kappa (2.9%) variants.

Conclusion

Although the vaccination does not restrict/avoid infection, it appears to protect the vaccinated people from severe forms of COVID-19. Also, the higher Ct values among vaccinated people indicate that the viral load among such people may be lower and, therefore, minimizes viral transmission.

## Introduction

Ever since the beginning of the COVID-19 pandemic, we have been looking for measures to control the spread of the virus and minimize morbidity and mortality. Developing the vaccine against the novel SARS-CoV-2 virus was one of the major milestones achieved in limiting the spread of the virus. Various vaccines have been developed and used worldwide. Moreover, vaccinating people can be an effective way of controlling the ongoing COVID-19 pandemic because vaccines have shown high efficacy in preventing serious illness and hospitalization. However, the long-term effectiveness of the vaccines is still unknown [1,2]. Vaccines are not fully effective as evidenced by the reports of fully vaccinated people getting COVID-19 infection. An infection of a completely vaccinated individual is called a vaccine breakthrough infection. Available literature indicates that breakthrough infections generally have mild symptoms, fewer complications, and low hospitalization rates [3]. Although the viral load may be the same or even higher in the case of vaccine breakthrough infection, it generally declines rapidly. However, such individuals remain infected and contagious for a shorter period. The increase in the number of infection rates, especially with the Delta variant and the newly emerged Omicron variant, has made it clear to both healthcare workers (HCWs) and the general population that even fully vaccinated individuals are also at risk of getting SARS-CoV-2 infection. Therefore, in July 2021 the US Center for Disease Control and Prevention (CDC) modified its guidelines for fully vaccinated people. The CDC advised all the communities who were staying in the area with high viral loads or high transmission rates should wear the masks even indoors irrespective of their vaccination status [4]. There are several ongoing studies to find out ways for diagnosing the vaccine breakthrough infection using genomic studies to know about the variants and the correlation of vaccination with clinical outcomes [3]. Understanding vaccine breakthrough infections is essential to appropriately tackle the ongoing pandemic. This study was carried out to evaluate SARS-CoV-2 breakthrough infections and compare the clinical profile and outcomes of the vaccinated and unvaccinated populations. 

## Materials and methods

A retrospective observational study was conducted at Shri B.M. Patil Medical College Hospital and Research Centre during March and April 2021 (2 months). A total of 103 patients have been included in the study who reported during the study period. The inclusion criteria were all the fully vaccinated and unvaccinated patients who tested positive for SARS-CoV-2 infection were included in the study. The people who were partially vaccinated were excluded from the study. Informed consent was obtained from all the study subjects and the study was approved by the Institutional ethics committee (BLDE (DU)/IEC/567/2021-22). 

Age, gender, occupation, COVID-19 profile (symptoms and hospitalization status), computed tomography severity score (CT-SS), the COVID-19 reporting and data system (CO-RADS) score, clinical outcome, vaccination status, and type of vaccine were collected from the patient's medical records. Further, COVID-19-positive samples were screened for lineages/variant/strain identification using next-generation sequencing (NGS). 

CO-RADS system of assessing clinical severity

The CO-RADS scoring system was applied and interpreted as suggested by Fujioka et al. [5]. The CO-RADS scores of 1, 2, 3, 4, 5, and 6 were interpreted as not interpretable, scan technically insufficient for assigning a score; very low normal or noninfectious; low typical for other infections but not COVID-19; equivocal/unsure features compatible not only with COVID-19 but also other diseases; highly suspicious of COVID-19; very highly typical for COVID-19; and proven positive for SARS-CoV-2 infection on reverse transcription polymerase chain reaction (RT-PCR), respectively.

CT-SS system of assessing the severity 

CT-SS was measured based on the extent of the involvement of the lung lobes as suggested by Hu et al. and Bernheim et al [6,7]. The CT-SS scores of 1, 2, 3, 4, and 5 were interpreted as a lung involvement of 0-5%; 5-25%; 25-50%; 50-75%; and 75-100%, respectively.

Data were entered into Microsoft Office 2019 Excel sheets (Microsoft® Corp., Redmond, USA) and analyzed using Statistical Package for Social Sciences (SPSS) version 24.0 (IBM Corp., NY, USA), The data were presented as percentages, means, and standard deviation.

## Results

The mean age of study participants was 46.22±17.77 years. Of the patients included, 64 (62.1%) were males and 39 (37.9%) were females. 11 (10.7%) were HCWs and 92 (89.3%) were non-HCWs. 33 (32%) were vaccinated and 70 (68%) were unvaccinated. Out of 33 vaccinated people, 10 (9.7%) were vaccinated with COVAXIN® (Inactivated, BBV152, Bharath Biotech) and 23 (22.3% ) were vaccinated with COVISHIED^TM^ (mRNA, AZD1222 (ChAdOx1), Oxford/AstraZeneca). The days since completing the vaccination and returning a positive result among the vaccinated participants was 37.09±23.74 days. Among the study participants, only 30 (29.2%) were hospitalized due to COVID-19. The mean Cycle threshold (Ct) values of the total study participants observed in terms of the amplified gene during the PCR among total participants (E Gene/N Gene: 18.84±4.898, RdRp/S Gene: 20.48±5.604), the vaccinated group (E Gene/N Gene: 22±4.25, RdRp/S Gene: 24.5±4.35), and the unvaccinated study group (E Gene/N Gene: 17.38±4.53, RdRp/S Gene: 18.57±5.14) varied significantly. 76.7% of the study participants were infected with Delta (B.1.617.2) variant followed by 20.4% with Delta (AY.4), and 2.9% with Kappa.

Most of the symptomatic patients suffered episodes of fever, headache, sore throat, fatigue, cough, and breathlessness. As many as 30 (37.9%) symptomatic patients required hospitalization for appropriate management. 18 (60%) hospitalized patients succumbed to the disease. The average CO-RAD score of the patients who could not survive (18.09±6.02) was significantly higher than those who recovered (5.27±2.86) after the hospitalization. The details of the symptomatology, vaccination status, and the virus variant responsible are listed in Table 1.

**Table 1:** The symptomatology, vaccination status, the virus variant responsible for the infection, and the clinical outcomes among the study participants CT-SS: Computed tomography severity score; CO-RADS: COVID-19 reporting and data system

Parameter	Variable	Number of Individuals (Total=103); n (%); Mean±SD
Sex	Male	64 (62.1%)
	Female	39 (37.9%)
Vaccination status	Vaccinated	33 (32%)
	Unvaccinated	70 (68%)
Time to positivity after complete vaccination	Vaccinated	37.09±23.74
Variant of virus	Delta (AY.4)	21 (20.4%)
	Delta (B.1.617.2)	79 (76.7%)
	Kappa (B.1.617.1)	3 (2.9%)
Symptomatic status	Symptomatic	79 (76.7%)
	Asymptomatic	24 (23.3%)
Hospitalization	Yes	30 (37.9%)
	No	49 (62%)
Mortality among hospitalized patients	Yes	18 (60%)
	No	12 (40%)
CT-SS score	Dead	18.09±6.02
	Recovered	5.27±2.86
CO-RADS score	Dead	5
	Recovered	5

Among 33 vaccine breakthrough infections, 20 (60.6%) were asymptomatic, 13 (39.4%) were symptomatic and no deaths were reported. Of the 70 unvaccinated patients, 18 patients (25.7%) succumbed, and 52 patients (74.3%) recovered. Of the 70 unvaccinated patients 94.2% were symptomatic and only 5.8% remained asymptomatic. The details of the vaccination status, symptomatology, recovery, and mortality are depicted in Table 2.

**Table 2:** The vaccination status, Ct values, symptomatology, recovery, and mortality among study participants Ct: Cycle threshold

Status of vaccination (n)	Ct values (Mean±SD)		Symptomatic	Asymptomatic	Death	Recovered
	E Gene/N Gene	RdRp/S Gene				
Vaccinated (33)	22 ±4.25	24.5 ±4.35	13 (39.4%)	20 (60.6%)	0 (0%)	33 (100%)
Unvaccinated (70)	17.38 ±4.53	18.57 ±5.14	66 (94.2%)	4 (5.8%)	18 (25.7%)	52 (74.3%)
All participants (103)	18.84 ±4.898	20.48 ±5.604	79 (76.7%)	24 (23.3%)	18 (17.4%)	85 (82.5%)

## Discussion

The COVID-19 pandemic caused by the novel SARS-CoV-2 has now been in existence for more than two years after its discovery on the Chinese mainland in November 2019 [8]. COVID-19 has caused extensive social and financial damages globally, equally involving both developed and developing nations [9]. The severity of the disease left millions of people dead, and several others have suffered extreme morbidity. Despite the availability of a vaccine, we have not yet completely gotten rid of the virus owing to its repeated genetic variations and development into variants of concern [10,11]. This has resulted in a situation wherein vaccinated people are susceptible to infection with the virus. Such infections among the completely vaccinated population are termed breakthrough infections [12,13]. In the present study, we have noted that the vaccination was effective in minimizing the mortality attributed to the infection despite it being ineffective in completely restricting the infection. Most vaccinated people had higher Ct values as compared to the unvaccinated subjects highlighting the fact that the vaccines could lower the viral load and thereby minimize the viral transmission. Given the absence of deaths among the vaccinated people, it can be concluded that the vaccines may still be effective in terms of minimizing the severity of the disease.

As compared to the general population, HCWs are at increased risk of vaccine breakthrough infection due to prolonged and repeated exposure to hospitalized patients. About 27% of HCWs contracted COVID-19 despite being fully vaccinated [14,15]. In a country like India, where most geographical regions are densely populated, vaccine breakthrough infections may potentially occur among the general population as noted in our study.

The vaccine effectiveness (BNT162b2, Pfizer/BioNTech) after complete vaccination among people with severe comorbidities was assessed in an Israeli study. COVID-19 patients with cardiovascular comorbidities, chronic lung, and kidney disease patients, and cancer patients, among others suffered 22% mortality despite full vaccination [16].

The vaccine breakthrough infections are not specific to any particular viral variant. This is evident by the observations of a study from the United States of America (USA). It was noted that the fully vaccinated people who had taken the mRNA vaccines (BNT162b2 (Pfizer/BioNTech), mRNA-1273 (Moderna), or JNJ-78436735 (Janssen)) suffered from breakthrough infections with the B.1.1.7 (Alpha) or B.1.526 (Iota) variants of SARS-CoV-2 [17]. Therefore, it is evident that breakthrough infections have multiple factors and are not attributed to any specific variant of the virus.

The antibody titers after the second vaccination dose peak after a month and later drop down drastically. This was noted among people who were fully vaccinated with the BNT162b2 (Pfizer/BioNTech) mRNA vaccine [18]. This study signifies the importance of the measurement of antibody titers before a decision on booster doses.

The B.1.1.7 (alpha) variant was found in the majority of the breakthrough infections among the HCWs who had received the BNT162b2 (Pfizer/BioNTech) mRNA vaccine. This study noted that there was a correlation between the concentrations of neutralizing antibodies and the occurrence of breakthrough infections [19]. More than two-fold risk of infection was noted among the people who had just completed the vaccination with BNT162b2 (Pfizer/BioNTech) mRNA vaccine and among those who had been completely vaccinated four months prior [10]. This study highlights the fact that immunity wanes over time and therefore further studies may still be needed to confirm the real-time protection conferred by the current vaccines. Vaccine breakthrough infection after complete vaccination with the Pfizer BNT162b2, Moderna mRNA-1273, and Covaxin BBV152 vaccines showed that the Delta variant is more efficient in terms of immune escape as compared to the Alpha, Iota, and other variants. Therefore, there is an increased possibility of vaccine breakthrough infections related to the Delta variant [2].

Infection with the Delta variant after 10 days of complete vaccination was reported among patients who received the Covaxin BBV152 vaccine. The patients suffered from mild to moderate symptoms. However, a couple of patients succumbed to the infection after a month in the Intensive care unit management and the Delta variant was found associated with these infections [20]. This suggests that the concentrations of the neutralizing antibodies after complete vaccination were inadequate to protect against severe infection and death against the Delta variant infection. 

Assessment of COVID-19 among hospitalized patients revealed that a CT-SS greater than 7.5, and a CO-RADS score of more than 5 may correspond to increased severity and poor clinical outcome [21]. In the present study, we observed that all hospitalized patients had a CO-RAD score of around 5. However, the CT-SS of the patients who succumbed to COVID-19 was significantly higher than those who recovered. This observation suggests that the CT-SS may be more beneficial in assessing the clinical severity of COVID-19.

The mean CT-SS scores after infection with SARS-CoV-2 among the vaccinated and unvaccinated people were found significantly different. The vaccinated patients had lower CT-SSs (3.5±6.3) as compared to the unvaccinated people (10.1 ± 11.4). It was also noted that the vaccinated people had higher Ct (Ct>20) values as compared to the unvaccinated people (Ct<20) [22]. These observations were corresponding to the results obtained from our study. Minimal clinical severity as depicted by the lower CT-SSs among the vaccinated people as compared to the unvaccinated population was also reported by other studies [23-25].

Limitations

A major limitation of this study was the inclusion of people who were recently vaccinated and the participants were not evaluated based on their comorbidities. Moreover, it was not possible to evaluate the long-term efficacy of vaccines. Given that the currently prevailing virus variants include the Omicron and its variety of sublineages, this study does not evaluate the breakthrough infections among such cases.

## Conclusions

The results of the current study support the fact that the emergence of the Delta variant was responsible for the second wave of infection and resulted in several breakthrough infections in India. Moreover, the clinical outcomes were significantly different among the vaccinated and unvaccinated people. It appears that maximum protection from the vaccination may take more than one month after the second dose. Most unvaccinated people developed symptoms and many of them suffered a severe form of COVID-19 as evidenced by the lower Ct values and higher CT-SSs. All the COVID-19-related deaths were noted among unvaccinated people. Vaccinated people developed either asymptomatic or only suffered minor illnesses and did not require hospitalization. Despite the emergence of viral variants of concern showing increased mutations including the Omicron, the vaccine may still be effective and could be protecting people against severe illnesses. Therefore, it is suggested that breakthrough infections may be inevitable, and large-scale vaccine coverage, assessment of neutralizing antibody concentrations among the vaccinated people, deciding on the requirement for booster doses, and development of improved vaccines effective against novel viral variants may be seriously considered in future studies.
